# Complementary/Alternative versus Prescription Medications

**DOI:** 10.15844/pedneurbriefs-29-12-5

**Published:** 2015-12

**Authors:** J. Gordon Millichap

**Affiliations:** 1 Division of Neurology, Ann & Robert H. Lurie Children's Hospital of Chicago, Chicago, IL; 2 Departments of Pediatrics and Neurology, Northwestern University Feinberg School of Medicine, Chicago, IL

**Keywords:** Complementary and Alternative Medicine, Headache, Chronic Fatigue, Epilepsy

## Abstract

Investigators from the Mayo Clinic, Rochester MN, determined the use of complementary and alternative medicine (CAM) in an outpatient pediatric neurology clinic, and assessed family attitudes toward the efficacy of CAM versus prescription medications.

Investigators from the Mayo Clinic, Rochester MN, determined the use of complementary and alternative medicine (CAM) in an outpatient pediatric neurology clinic, and assessed family attitudes toward the efficacy of CAM versus prescription medications. Questionnaires distributed to 500 consecutive patients alluded to the child's diagnosis, use of CAM, efficacy of CAM and the prescription medication. Of 484 surveys returned, 327 were usable. Only 17.4% admitted initially to the use of CAM to treat neurological problems. At follow-up, 41.6% of patients understood that they used CAM. Disorders treated with a significant increased prevalence of CAM included headache (50.8% with headache used CAM vs 35.7% without headache, p=0.008), chronic fatigue (63.2% vs 38.8%, p=0.005), and sleep disorders (77.1% vs 37.3%, p<0001). Epilepsy was among disorders least likely to be treated with CAM (107/327, 32.7%), Melatonin was the most widely used CAM, followed by probiotics. Gluten-free diet was among the top 10 popular modalities. Only 38.5% of CAM using patients recognize that they are taking CAM, a finding that demonstrates the need to inquire in depth about use of CAM. Patients less satisfied with their prescription medications are more likely to use CAM, reflecting the less tractable nature of their disorders. Few families (4.9%) managed to treat solely with CAM. [1]

COMMENTARY. The Mayo Clinic, renown for its allopathic medicine, must be congratulated on a detailed study of the prevalence of use and perceived efficacy of complementary and alternative medicine (CAM). The list of 56 commonly used CAM modalities showing their frequency of use is particularly of interest and emphasizes the popularity of melatonin, probiotics, music therapy, and omega 3 among parents of patients in this particular clinic. While different communities may demonstrate different patterns of use of CAM, the educational level did not affect the use of CAM in this community. Most pediatric CAM use is not discussed with patients, despite the interest frequently shown by parents [2]. The authors note that patients are less likely to report the use of CAM unless asked about specific modalities. Making the Mayo Clinic list of commonly used CAM modalities available to patients should facilitate the physician-parent discussion and lead to a better understanding of the indications and need for evaluation of this form of therapy. For headache, the most frequently treated disorder using CAM, long-term prophylactic drug therapy is appropriate only after exclusion of headache precipitating trigger factors, including dietary factors [3].
